# Nanomaterial‐Enhanced Biosensing: Mechanisms and Emerging Applications

**DOI:** 10.1002/adhm.202500189

**Published:** 2025-04-01

**Authors:** Younghak Cho, Yunyoung Choi, Yerim Jang, Hyejeong Seong

**Affiliations:** ^1^ Brain Science Institute Korea Institute of Science and Technology (KIST) Seoul 02791 Republic of Korea; ^2^ Department of Chemical and Biomolecular Engineering Korea Advanced Institute of Science and Technology (KAIST) Daejeon 04141 Republic of Korea; ^3^ Division of Bio‐Medical Science and Technology KIST School Korea University of Science and Technology (UST) Seoul 02791 Republic of Korea

**Keywords:** biosensors, diagnostics, nanomaterials, nanostructures, point‐of‐care

## Abstract

Biosensors serve as indispensable analytical tools in biomedical diagnostics, environmental monitoring, and personalized healthcare, offering operation simplicity, cost‐effectiveness, high sensitivity, and portability. Nanostructure integration has overcome traditional sensing platform limitations, particularly in sensitivity and response dynamics. These nanoscale materials—including nanoparticles, nanowires, nanosheets, and nanotubes—leverage unique physicochemical properties such as high surface‐to‐volume ratio, quantum confinement effects, and plasmonic interactions to enhance biosensor performance significantly. This review systematically analyzes recent advances in nanostructure‐based biosensing technologies, examining how nanomaterial engineering improves sensor sensitivity, selectivity, and multifunctionality. Fundamental mechanisms are explored by which nanostructures enhance electrochemical, optical, and electrical biosensor performance, emphasizing low‐abundance biomarkers in complex biological matrices. Beyond technological innovations, practical applications are evaluated across healthcare and environmental monitoring. Finally, current challenges and outline future research directions, highlighting these technologies' potential are addressed to transform diagnostic capabilities and healthcare outcomes.

## Graphical abstract

Nanomaterial integration transforms biosensor capabilities through enhanced signal transduction, sensitivity, and selectivity. This review analyzes how nanoscale materials—from nanoparticles to nanosheets—leverage unique physicochemical properties to revolutionize electrochemical, optical, and electrical biosensing. Current challenges and future directions are examined, highlighting nanomaterial‐enhanced biosensors' potential to transform healthcare and environmental monitoring.

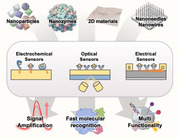

## 1 Introduction

Biosensors have emerged as transformative analytical tools, offering exceptional advantages through operational simplicity, cost‐effectiveness, high sensitivity, portability, and straightforward construction. These devices integrate biological recognition elements with electronic components, enabling broad applications across multiple fields.^[^ ^1^ ^]^ Various biosensing technologies—including amperometric sensors, electrochemical impedance sensors, luminescence sensors, and photoelectrochemical sensors— have achieved widespread use in detecting chemical and biological targets through precise measurements of dynamic changes at electrode interfaces.^[^ ^2^ ^]^

Nanotechnology integration has revolutionized biosensors capabilities. Nanoscale materials, such as nanoparticles, nanowires, nanosheets, nanoneedles, and nanotubes, significantly enhance sensor performance through improved signal transduction,^[^ ^3^ ^]^ heightened sensitivity,^[^ ^4^ ^]^ enhanced selectivity,^[^ ^5^ ^]^ lower detection limits, and accelerated response dynamics.^[^ ^6^ ^]^ These advances have expanded applications beyond traditional biomedical research into clinical diagnostics^[^ ^7^ ^]^ and personalized healthcare, enabling previously undetectable target detection.

**Figure 1:**
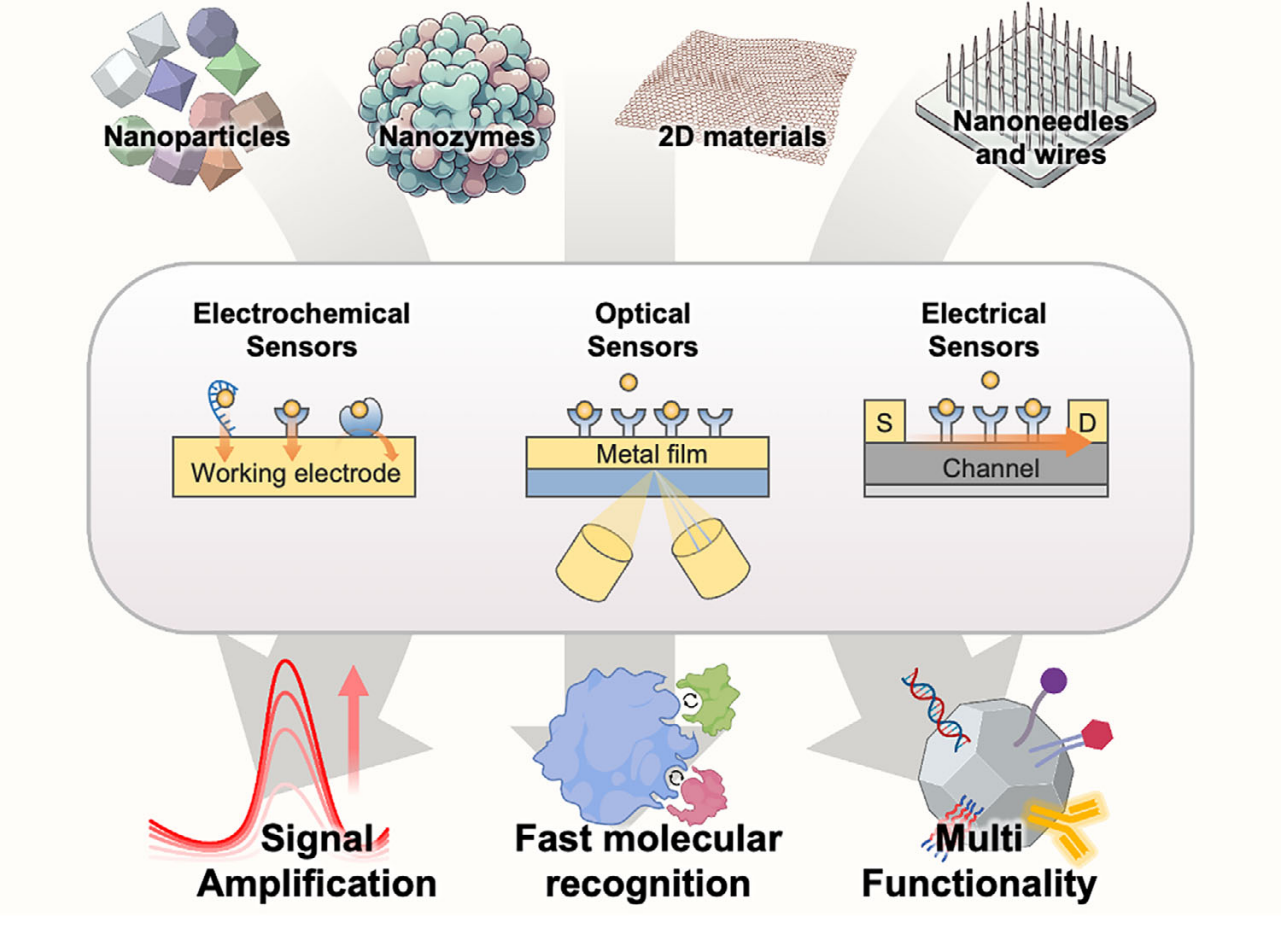
Nanostructure‐enhanced biosensing: Illustration of nanostructures, including nanoparticles, nanozymes, 2D materials, and nanoneedles/wires, integrated into electrochemical, optical, and electrical biosensors. These nanostructures enhance signal amplification, sensitivity, and multifunctionality, driving advancements in biosensor performance and application diversity.

This review examines recent advances in nanostructure‐based biosensors, focusing on nanomaterials science and engineering contributions to biosensor functionality (**Figure** **1**). We aim to provide systematic analysis of nanostructure‐based biosensing technologies, emphasizing mechanisms enhancing sensor performance. Our analysis highlights emerging technologies' potential to transform healthcare and environmental diagnostics. Additionally, we address current challenges in nanostructure‐incorporated biosensing and propose promising future research directions. While comprehensive, we acknowledge this dynamic field's vast scope necessitates selective coverage, and we extend our apologies to researchers whose valuable contributions may not be included in this review.

## 2 Properties of Nanomaterials in Biosensors

### 2.1 Nanomaterial Properties

Nanomaterials, with dimensions typically ranging from 1 to 100 nm, exhibit distinct physical and chemical properties fundamentally different from their bulk counterparts. Understanding these characteristics is essential for optimizing their integration into biosensing platforms. This section summarizes the key characteristics that enable nanomaterials to enhance signal amplification and molecular recognition in biosensor applications.

#### 2.1.1 Size Effect

The size effect represents one of the most fundamental principles governing nanomaterial behavior, characterized by high densities of surface defects, grain boundaries, crystalline phase interfaces, and pores. These structural characteristics significantly influence their mechanical, electrical, optical, chemical, and magnetic properties compared to bulk materials.

**Figure 2:**
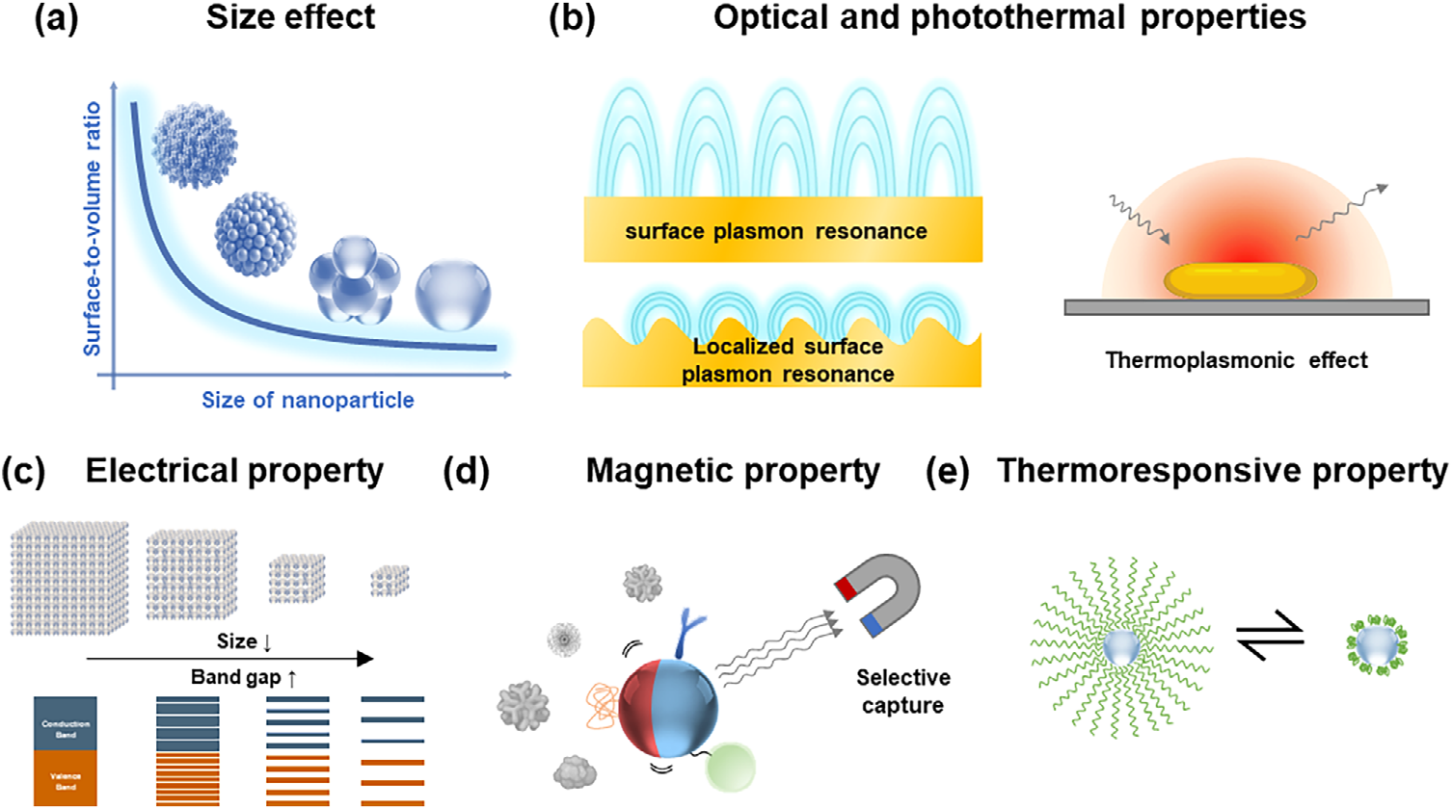
Key properties of nanomaterials that enhance biosensing performance. a) The relationship between nanoparticle size and surface‐to‐volume ratio, demonstrating increased surface area with decreasing size. b) Illustration of LSPR occurring in nanostructures and localized heat generation by thermoplasmonic effect. c) Size‐dependent electrical properties of nanostructures, highlighting the widening bandgap as material dimensions decrease from bulk to nanoscale, driven by quantum confinement effects. d) Functionalized magnetic nanoparticles selectively capturing target biomolecules from complex biological fluids using an external magnetic field, enhancing detection sensitivity. e) Thermoresponsive effect of PNIPAM‐functionalized nanoparticle.

The increased surface area‐to‐volume ratio^[^ ^8^ ^]^ provides the most significant advantage, dramatically enhancing binding capacity for target biomolecules (**Figure** **2**a). This expanded surface area provides numerous active sites for molecular interaction, substantially improving the sensor sensitivity. Various nanostructures—including nanoparticles,^[^ ^9^ ^]^ nanowires,^[^ ^10^ ^]^ nanoneedles,^[^ ^11^ ^]^ and nanotubes^[^ ^12^ ^]^—provide extensive surfaces for functionalization with specific receptors or probes, enabling improved analyte capture and detection. This property is especially crucial for early‐stage disease diagnostics.

#### 2.1.2 Optical and Photothermal Properties

Nanomaterials demonstrate distinctive optical properties arising from quantum confinement, plasmonic resonance, and enhanced light‐matter interactions. Metallic nanoparticles, particularly gold (Au) and silver (Ag), support localized surface plasmon resonance (LSPR),^[^ ^13^ ^]^ where incident light induces collective oscillations of free electrons. This phenomenon enhances electromagnetic fields at the nanoparticle surfaces, amplifying optical signals in detection techniques such as surface‐enhanced Raman spectroscopy (SERS) (Figure 2b).^[^ ^14^ ^]^ These properties enable highly sensitive optical biosensors capable of detecting picomolar analyte concentrations,^[^ ^15^ ^]^ advancing applications from molecular imaging to pathogen detection.

Quantum dots (QDs) demonstrate size‐dependent optical properties due to quantum confinement, with emission wavelengths shifting toward shorter wavelengths as particle size decreases, following:

(1) $$\lambda =\frac{hc}{\Delta E}$$

where λ represents light wavelength, ℎ is Planck's constant, 𝑐 is light speed, and *ΔE* represents the energy difference between electronic states. This relationship enables precise spectral tuning for fluorescence‐based biosensing and multiplexed detection.^[^ ^16^ ^]^

Metallic nanostructures also exhibit valuable photothermal properties. When irradiated at plasmon resonance frequencies, they efficiently convert absorbed light into localized heat through the thermoplasmonic effect (Figure 2b).^[^ ^17^ ^]^ This localized heating accelerates biomolecular interactions and reduces non‐specific binding, enabling label‐free detection. Thermoplasmonic biosensors have been widely used to detect nucleic acids, proteins, and pathogenic microorganisms by leveraging heat‐induced binding kinetics.^[^ ^18^ ^]^

#### 2.1.3 Electrical Properties

The electrical properties of nanomaterials, such as conductivity and resistivity, undergo significant changes at the nanoscale. When dimensions decrease below the electron mean free path, surface scattering increases resistivity,^[^ ^19^ ^]^ while energy level confinement can transform semiconductors into insulators and metals into semiconductors (Figure 2c). These characteristics enable development of energy‐efficient, flexible electronic sensors.^[^ ^20^ ^]^

Graphene exemplifies these properties through its two‐dimensional (2D) sp^2^‐hybridized carbon structure. Its single‐atom‐thick honeycomb lattice produces extraordinary electron mobility and conductivity compared to bulk carbon.^[^ ^21^ ^]^ Similarly, carbon nanotubes (CNTs) and nanowires demonstrate remarkable properties such as ballistic conduction^[^ ^22^ ^]^ and low resistivity, enhancing signal transduction in resistive biosensors.^[^ ^23^ ^]^

MXenes, a family of 2D metal nitrides and carbides, combine high electrical conductivity with hydrophilic surfaces.^[^ ^24^ ^]^ This dual functionality makes them particularly valuable for electrochemical biosensing platforms.

#### 2.1.4 Magnetic Property

Magnetic nanomaterials enhance biosensing through controlled manipulation, separation, and signal amplification.^[^ ^25^ ^]^ Superparamagnetic nanoparticles, particularly iron oxide (Fe_3_O_4_), exhibit high magnetic susceptibility without retaining magnetization after field removal.^[^ ^26^ ^]^ These materials enable selective capture of target biomolecules from complex biological fluids using an external magnetic field, improving detection sensitivity through reduced background noise (Figure 2d).^[^ ^27^ ^]^

When functionalized with specific recognition elements, magnetic nanoparticles bind target analytes for rapid isolation before detection.^[^ ^28^ ^]^ They also enhance biosensing through magnetoresistive effects and magnetic relaxation processes. Giant magnetoresistance sensors detect changes in electrical resistance from magnetically labeled biomolecules, achieving femtomolar detection limits.^[^ ^29^ ^]^

Additionally, magnetic particle relaxation biosensors offer label‐free, real‐time biosensing by monitoring biomolecule binding through rotational behavior changes under oscillating magnetic fields.^[^ ^30^ ^]^

#### 2.1.5 Thermoresponsive and Other Properties

Thermoresponsive behavior and thermal characteristics complement the fundamental advantages of nanomaterials in biosensing designs. These properties include specialized temperature‐dependent phase transitions and advanced heat‐transfer behavior at the nanoscale. For instance, polymer‐functionalized nanostructures demonstrate temperature‐dependent phase transitions that enable controlled biomolecular interactions.^[^ ^31^ ^]^ Poly(N‐isopropylacrylamide) (PNIPAM) exemplifies this behavior, exhibiting a hydrophilic‐to‐hydrophobic transition at ≈32 °C. Below this temperature, PNIPAM chains extend in a hydrophilic state; above the threshold, they collapse hydrophobically. This reversible transition enables dynamic biosensing applications, including temperature‐controlled molecular separation and signal modulation (Figure 2e).^[^ ^32^ ^]^

Certain nanomaterials exhibit exceptional thermal conductivity, facilitating heat dissipation and localized temperature control. Graphene and other 2D materials possess ultra‐high thermal conductivity, valuable for biosensing platforms requiring precise thermal management.^[^ ^33^ ^]^ Integration of thermal conductivity with optical and electrical transduction mechanisms enhances biosensor stability, reaction kinetics, and signal reliability.^[^ ^34^ ^]^

### 2.2 Signal Amplification Technique Using Nanomaterials

**Figure 3:**
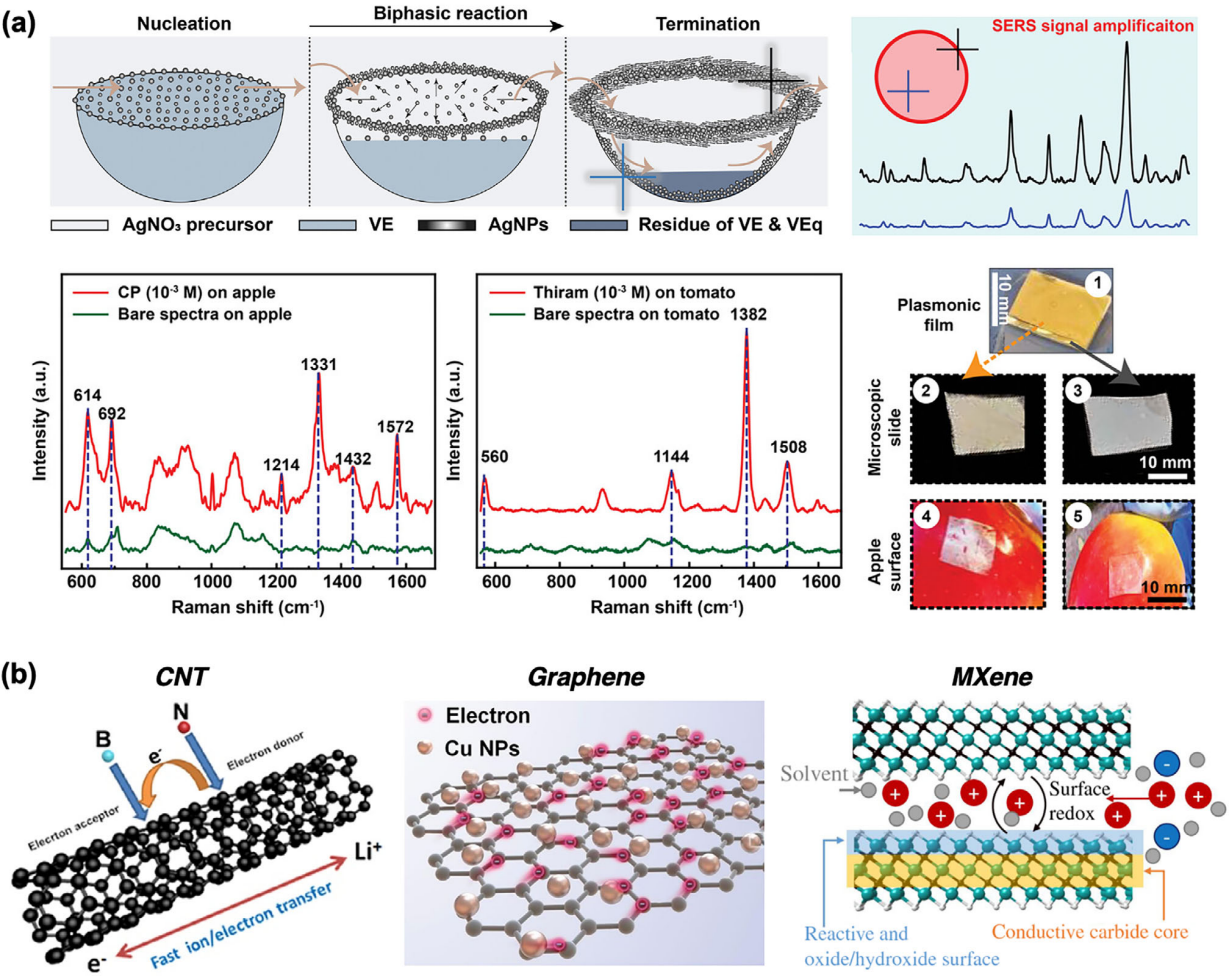
Nanostructure‐based signal amplification techniques in biosensing. a) Metallic nanoparticles and nanohole arrays enhance electromagnetic fields via LSPR, significantly boosting optical signals for sensitive detection on fruit surfaces. Reproduced with permission.^[^ ^37^ ^]^ Copyright 2020, Elsevier. b) Nanostructured materials such as CNTs, graphene, and MXene improve electrochemical biosensors by increasing electron transfer rates and conductivity, enabling the detection of biomolecules at ultra‐low concentrations. Reproduced with permission.^[^ ^42^ ^]^ Copyright 2018, Elsevier. Copyright 2023, Elsevier. Copyright 2021, Springer Nature.

Signal amplification is crucial for improving biosensor detection limits,^[^ ^35^ ^]^ directly affecting early‐stage disease diagnosis. Recent advances demonstrate how tailored plasmonic nanostructures enable unprecedented sensitivity in biosensing applications.^[^ ^36^ ^]^ For instance, the functionalization of thin polydimethylsiloxane film with silver nanoparticles, achieved through controlled droplet reactions in micro‐well arrays, has yielded a plasmonic film capable of precise analyte localization (**Figure** **3**a).^[^ ^37^ ^]^ These conformable films enable highly sensitive SERS measurements on plant surfaces, achieving detection limits ranging from 10^−16^ to 10^−13^ M for multiplexed analyte detection.^[^ ^38^ ^]^

Signal amplification particularly impacts electrochemical sensors utilizing highly conductive nanomaterials such as CNTs,^[^ ^39^ ^]^ graphene,^[^ ^40^ ^]^ and MXene.^[^ ^41^ ^]^ Their superior conductivity enhances electron transfer rates and significantly increases current response upon analyte binding (Figure 3b).^[^ ^42^ ^]^ These properties enable biomolecule detection at extremely low concentrations, pushing the boundaries of biosensing applications,^[^ ^12^, ^41^ ^]^ as discussed in Section 4.

### 2.3 Selectivity and Specificity Improvements in Molecular Recognition

**Figure 4:**
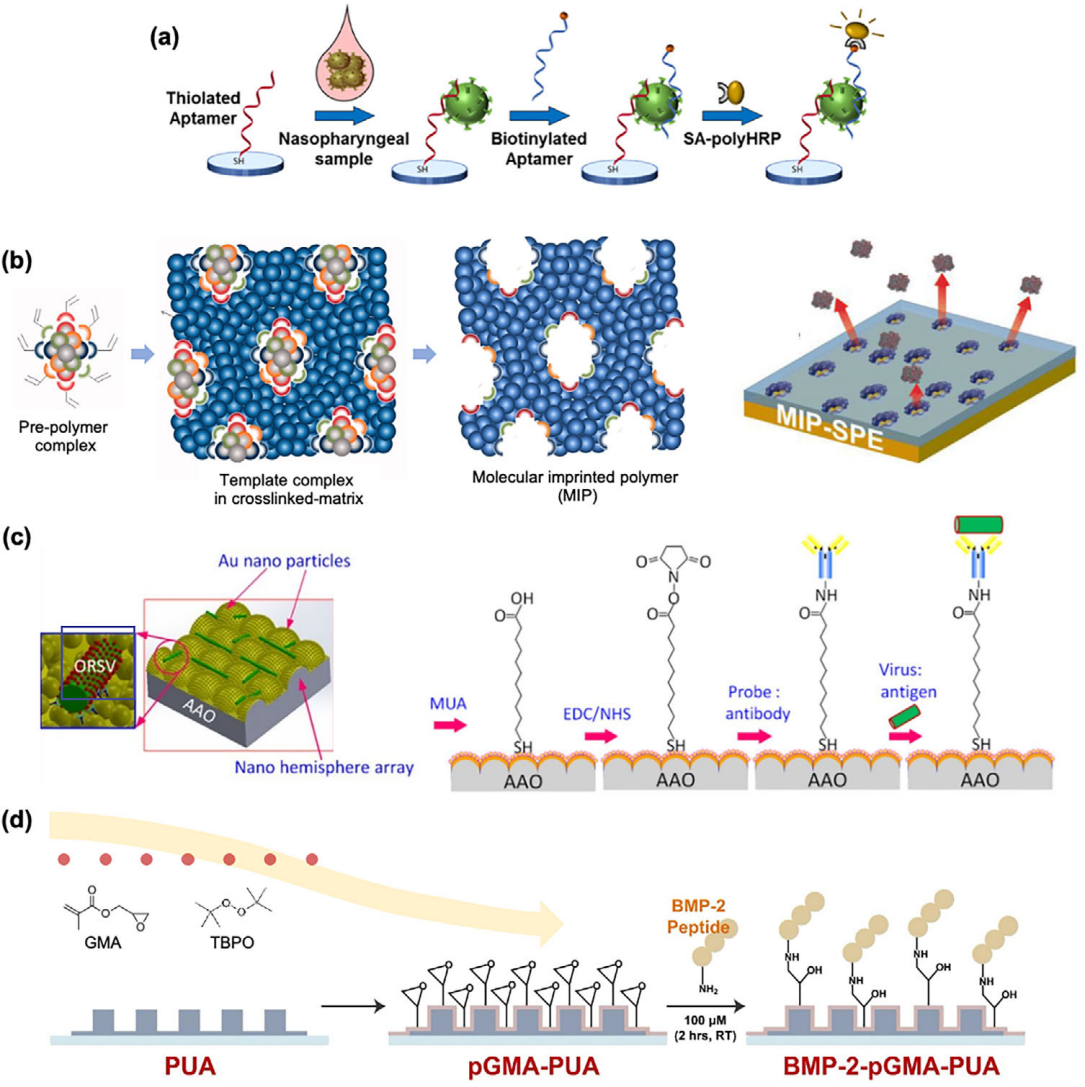
a) Advanced recognition mechanisms, such as sandwich assays and competitive binding formats, leverage nanomaterials to achieve high specificity, even in complex biological samples. Reproduced with permission.^[^ ^43^ ^]^ Copyright 2021, American Chemical Society. b) MIPs combined with nanostructures enable sensors with exceptional selectivity and resistance to cross‐reactivity, which are suitable for clinical, environmental, and food safety applications. Reproduced with permission.^[^ ^45^ ^]^ Copyright 2021, Springer Nature. Copyright 2022, Taylor and Francis. c,d) Functionalized nanostructures, created using techniques like SAM and iCVD, provide well‐organized surfaces for selective binding of target biomolecules. Reproduced with permission.^[^ ^64^ ^]^ Copyright 2018, Institute of Physics Science. Copyright 2013, Elsevier.

A biosensor's sensitivity —its ability to detect small changes in analyte concentration—is critical for accurate molecular recognition. Nanostructures substantially enhance sensitivity through improved molecular recognition capabilities in biosensing platforms. For example, biosensors utilizing nanomaterials can employ sandwich assays or competitive binding formats that are highly selective for specific biomolecules.^[^ ^15b,d^ ^]^ In sandwich assays, nanoparticles functionalized with secondary antibodies or aptamers increase binding capacity and act as amplification labels, boosting detection signals by orders of magnitude (**Figure** **4**a).^[^ ^43^ ^]^ Such approaches prove particularly valuable for detecting low‐abundance targets in complex matrices.

The integration of molecularly imprinted polymers (MIPs) with nanostructures creates highly selective sensors resistant to cross‐reactivity.^[^ ^44^ ^]^ These MIPs serve as robust alternatives to enzymatic recognition elements by providing precisely tailored binding environments for specific analytes (Figure 4b).^[^ ^45^ ^]^ Such hybrid systems demonstrate reliable performance across clinical diagnostics,^[^ ^46^ ^]^ environmental monitoring,^[^ ^47^ ^]^ and food safety testing.^[^ ^48^ ^]^

### 2.4 Multifunctionality in Biosensors

Nanomaterial‐based biosensors have evolved beyond single‐purpose detection to incorporate multiple integrated functionalities, expanding their practical applications across diverse fields. Surface chemistry modification represents a fundamental approach to achieving multifunctionality. By functionalizing nanostructures with various biological moieties—including antibodies,^[^ ^49^ ^]^ nucleic acids,^[^ ^50^ ^]^ and enzymes^[^ ^51^ ^]^—these platforms enable simultaneous detection of multiple biomarkers.^[^ ^52^ ^]^ This capability proves essential for comprehensive diagnostics, supporting rapid screening and early disease detection. The precise control of surface chemistry ensures selective binding of target biomolecules while minimizing non‐specific interactions,^[^ ^53^ ^]^ maintaining high sensitivity even in complex biological matrices where competing interactions typically compromise detection accuracy.^[^ ^54^ ^]^ Advanced surface modification techniques, such as self‐assembled monolayers (SAMs)^[^ ^55^ ^]^ and initiated chemical vapor deposition (iCVD),^[^ ^56^ ^]^ create well‐defined, organized surfaces that promote targeted interactions (Figure 4c,d). These approaches significantly enhance signal‐to‐noise ratios,^[^ ^57^ ^]^ improving overall biosensor performance.

Recent advances have expanded multifunctionality through integrated system capabilities. Modern biosensors incorporate sophisticated readout mechanisms and wireless communication, enabling continuous, real‐time health monitoring. Wearable platforms with flexible and stretchable electronics demonstrate this advancement by simultaneously tracking multiple physiological parameters, including sweat biomarkers^[^ ^58^ ^]^ and cardiac signals,^[^ ^59^ ^]^ while transmitting data wirelessly for immediate analysis.

Multi‐modal biosensing represents another key advancement. Platforms combining electrochemical, optical, and mechanical sensing modalities provide comprehensive diagnostics with improved reliability through signal cross‐validation.^[^ ^60^ ^]^ For instance, a recent study demonstrated a plasmonic Au nanostar@PtOs nanocluster (Au@PtOs) as a multi‐mode signal tag for lateral flow assay detection. By leveraging the PtOs bimetallic nanocluster doping strategy, Au@PtOs exhibited excellent SERS enhancement, nanozyme catalytic activity, and a superior photothermal effect compared to conventional approaches.^[^ ^61^ ^]^

Therapeutic capabilities further enhance biosensor multifunctionality. Smart platforms can trigger therapeutic responses based on detected biomarker levels, advancing closed‐loop therapeutic systems.^[^ ^62^ ^]^ These theragnostic approaches, combining diagnostic sensing with therapeutic delivery, show particular promise in personalized medicine applications.^[^ ^63^ ^]^

The integration of selective detection, real‐time monitoring, multi‐modal sensing, and therapeutic capabilities positions nanomaterial‐based biosensors as powerful tools for next‐generation biomedical applications, advancing precision medicine and real‐time bioanalysis.

## 3 Commonly Used Nanomaterials in Biosensing Platform

The advantages discussed in Section 2 arise from the unique properties of specific nanomaterials. These materials provide the foundation for advanced biosensing technologies, enabling enhanced sensitivity, specificity, and multifunctionality. This section explores the most prevalent nanomaterials in biosensing applications, examining their distinct contributions and the innovative platforms they enable.

### 3.1 Nanoparticles and Quantum Dots

**Figure 5:**
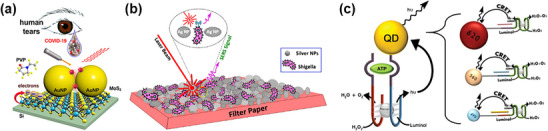
Applications of nanoparticles in biosensing. a) AuNPs in a SERS platform for COVID‐19 detection in human tears, enhancing sensitivity with 3D architectures. Reproduced with permission.^[^ ^68^ ^]^ Copyright 2024, American Chemical Society. b) AgNPs on filter paper for detecting Shigella bacteria, combining detection and antibacterial properties. Reproduced with permission.^[^ ^70^ ^]^ Copyright 2024, Springer Nature. c) Chemiluminescence resonance energy transfer with CdSe/ZnS QDs for sensitive luminescent biosensing. Reproduced with permission.^[^ ^72^ ^]^ Copyright 2011, American Chemical Society.

Metallic nanoparticles (NPs), particularly Au and Ag, are extensively employed due to their remarkable optical, electrical, and catalytic properties, as discussed in Section 2. AuNPs, renowned for their biocompatibility, ease of functionalization,^[^ ^65^ ^]^ and unique optical plasmonic properties, have been widely implemented in SERS^[^ ^15^, ^66^ ^]^ and LSPR,^[^ ^67^ ^]^ enabling ultra‐low biomolecule detection. Recent advancements demonstrate this capability through AuNP integration with SERS platform, exemplified by a biosensor developed for on‐site COVID‐19 detection using human tear samples (**Figure** **5**a).^[^ ^68^ ^]^ Close‐packed three‐demensional (3D)QD AuNP architectures significantly enhance SERS sensitivity, enabling sub‐terascale analyte detection. Similarly, AgNPs serve in biosensors targeting food safety and environmental monitoring, combining detection capability with strong antibacterial properties.^[^ ^69^ ^]^ For example, AgNP‐coated filter paper substrates function as highly sensitive SERS platforms for detecting *Shigella* bacteria, a significant cause of dysentery (Figure 5b).^[^ ^70^ ^]^

QDs complement metallic NPs in biosensing applications, offering size‐tunable fluorescence and photoluminescent properties for multiplexed analyte detection in single assays.^[^ ^71^ ^]^ Precise control of size and composition enables tunable emission wavelengths, facilitating simultaneous detection of multiple biomarkers through distinct fluorescence signals. The most studied QDs include cadmium‐based materials (e.g., CdSe, CdTe, and CdS), lead chalcogenides (e.g., PbS, PbSe), and ternary compositions (e.g., InP/ZnS), each with distinct bandgap energies determining optical performance. The versatile surface chemistry enables functionalization with biomolecules, including antibodies, nucleic acids, and peptides, enhancing selectivity and specificity.

QDs facilitate detection primarily through energy transfer processes, notably Förster resonance energy transfer (FRET) and chemiluminescence resonance energy transfer (CRET). A significant advancement involves CRET integration with CdSe/ZnS QDs,^[^ ^72^ ^]^ where specific biomolecular interactions generate highly sensitive luminescent signals (Figure 5c). This system employs a sophisticated DNAzyme comprising horseradish peroxidase fragments‐mimicking DNAzyme and ATP‐aptamer or Hg^2+^‐recognizing sequences. In the presence of ATP or Hg^2+^, the aptamer subunits and DNAzyme fragments self‐assemble into an active hemin–G‐quadruplex DNAzyme structure, efficiently catalyzing chemiluminescence.

### 3.2 Nanozymes

**Figure 6:**
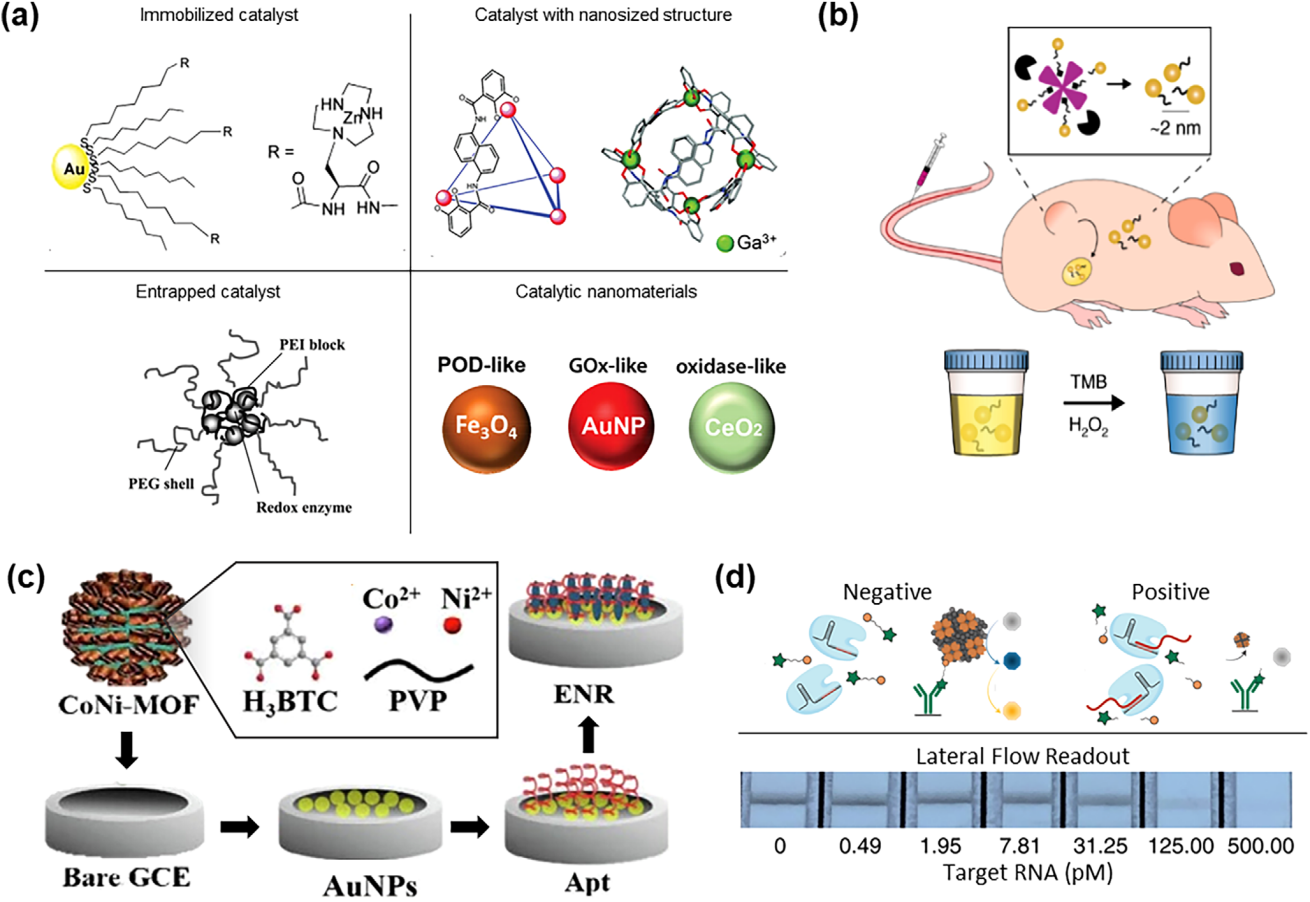
Nanozyme‐enhanced biosensing platforms for biomedical applications. a) Schematic representation of nanozymes catalyzing target analyte detection, significantly amplifying biosensor signals. Reproduced with permission.^[^ ^73e^ ^]^ Copyright 2024, Wiley. b) In vivo application of AuNC for cancer detection. Reproduced with permission.^[^ ^81^ ^]^ Copyright 2019, Springer Nature. c) CoNi‐based MOF with gold nanoparticles for ultrasensitive electrochemical aptasensor detection of enrofloxacin, useful in food safety. Reproduced with permission.^[^ ^83^ ^]^ Copyright 2022, Elsevier. d) Integration of nanozymes with CrisprZyme for quantitative RNA biomarker detection. Reproduced with permission.^[^ ^84^ ^]^ Copyright 2022, Springer Nature.

While NPs and QDs primarily enhance signals through optical and luminescent properties, nanozymes expand biosensing capabilities by introducing enzyme‐like catalytic functions that produce detectable signals (**Figure** **6**a).^[^ ^73^ ^]^ Various metal‐based nanozymes, derived from copper (Cu), nickel (Ni), cobalt (Co), and iron (Fe) and their oxides, demonstrate significant potential in their intrinsic catalytic activity.^[^ ^74^ ^]^ These materials exhibit multiple enzymatic behaviors, including peroxidase‐mimicking, oxidase‐mimicking, and catalase‐like activities, enabling efficient signal amplification in both electrochemical and colorimetric detection platforms. For instance, Fe_3_O_4_ NPs serve as peroxidase‐like nanozymes for biomarker detections,^[^ ^75^ ^]^ while Co‐based nanozymes show exceptional oxidase‐mimicking activity for rapid and selective biomolecule detection.^[^ ^76^ ^]^

Ultra‐small gold nanoclusters (AuNCs, 1–5 nm in size) offer exceptional biocompatibility for application in drug delivery,^[^ ^77^ ^]^ photothermal therapy,^[^ ^78^ ^]^ bioimaging,^[^ ^79^ ^]^ and biosensing.^[^ ^80^ ^]^ Their catalytic activity enables tumor‐specific enzymatic cleavage, producing colorimetric urinary readouts within 1 h. These nanosensors achieved a 13‐fold signal increase in tumor‐bearing mice with complete clearance within four weeks, demonstrating their potential for rapid, noninvasive diagnostics (Figure 6b).^[^ ^81^ ^]^

Metal‐organic frameworks (MOFs) have emrged as promising nanozyme platforms due to their high surface areas and tunable porosity.^[^ ^82^ ^]^ Combined with other nanomaterials, these hybrids enhance analyte binding sites, improving sensitivity and specificity. A notable example is a CoNi‐based MOF combined with AuNPs for ultra‐sensitive enrofloxacin detection (Figure 6c),^[^ ^83^ ^]^ achieving a detection limit of 3.3 × 10⁻⁴ pg mL^−1^ with excellent electrochemical stability.

Nanozymes show particular promise in advanced platforms such as CRISPR‐based diagnostics and lateral flow immunoassays. The CRISPR–Cas‐based diagnostic integrated with nanozyme‐linked immunosorbent assays (CrisprZyme) enables quantitative detection of RNA biomarkers associated with acute myocardial infarction and prostate cancer (Figure 6d).^[^ ^84^ ^]^ This platform combines CRISPR precision with nanozyme amplification for preamplification‐free, highly sensitive, colorimetric biomarker detection at room temperature.

### 3.3 2D Nanosheets: Graphenes and MXenes

2D materials—including graphene, MXene, and other graphene‐like materials such as transition metal dichalcogenides and hexagonal boron nitride (h‐BN) — exhibit exceptional electrical, optical, and mechanical properties for biosensing applications.^[^ ^85^ ^]^ Their atomically thin structures provide extraordinary high surface‐area‐to‐volume ratios, enabling efficient biomolecule immobilization and enhanced sensor‐analyte interactions. Their mechanical flexibility facilitates seamless integration into wearable and implantable biosensors, supporting scalable manufacturing and high‐throughput device fabrication. Their high electrical conductivity promotes rapid electron transfer, particularly advantageous for electrochemical biosensors, while their optical properties—including SPR and fluorescence quenching— contribute to highly sensitive optical sensing platforms.

**Figure 7:**
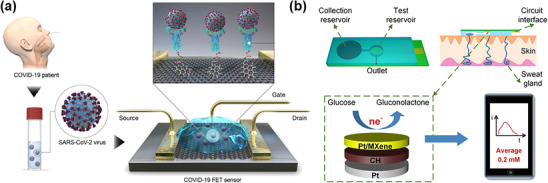
2D nanosheet‐based biosensing platforms. a) Graphene‐based FET biosensor functionalized with antibodies against SARS‐CoV‐2 spike proteins for rapid, label‐free detection in clinical samples. Reproduced with permission.^[^ ^86^ ^]^ Copyright 2020, American Chemistry Society. b) An MXene‐based flexible and wearable non‐enzymatic glucose sensor integrates Pt/MXene composites for continuous glucose detection in sweat. Reproduced with permission.^[^ ^87^ ^]^ Copyright 2023, American Chemistry Society.

Graphene‐based biosensors have demonstrated particular promise in clinical diagnostics through a field‐effect transistor (FET)‐based device for COVID‐19 detection (**Figure** **7**a).^[^ ^86^ ^]^ This platform, utilizing graphene sheets functionalized with SARS‐CoV‐2 spike protein antibodies, achieves detection at concentrations as low as 100 fg/mL in a clinical transport medium. This unprecedented sensitivity positions graphene FET‐based sensors as promising candidates for viral detection and pandemic preparedness.

MXene‐based biosensors advance wearable health monitoring through innovations such as flexible, non‐enzymatic glucose sensors incorporating Pt/MXene composites for continuous sweat glucose detection (Figure 7b).^[^ ^87^ ^]^ The hybridization of Pt NPs with Ti_3_C_2_T_x_ MXene nanosheets achieves a broad linear detection range (0–8 mmol L^−1^) under neutral conditions. The Pt/MXene catalyst, stabilized within a conductive hydrogel matrix, integrates with a microfluidic patch for efficient sweat collection. This system tracks glucose fluctuations correlating with energy metabolism, demonstrating patterns comparable to blood glucose levels. Recent development includes MXene/Prussian blue composites in stretchable, modular biosensors for comprehensive sweat analysis.^[^ ^41b^ ^]^ While MXenes offer significant advantages in biosensing, challenges regarding oxidation susceptibility and long‐term material stability require attention to ensure reliable and reproducible sensor performance.

### 3.4 High‐aspect‐Ratio Structures: Nanotubes, Nanowires, and Nanoneedles

**Figure 8:**
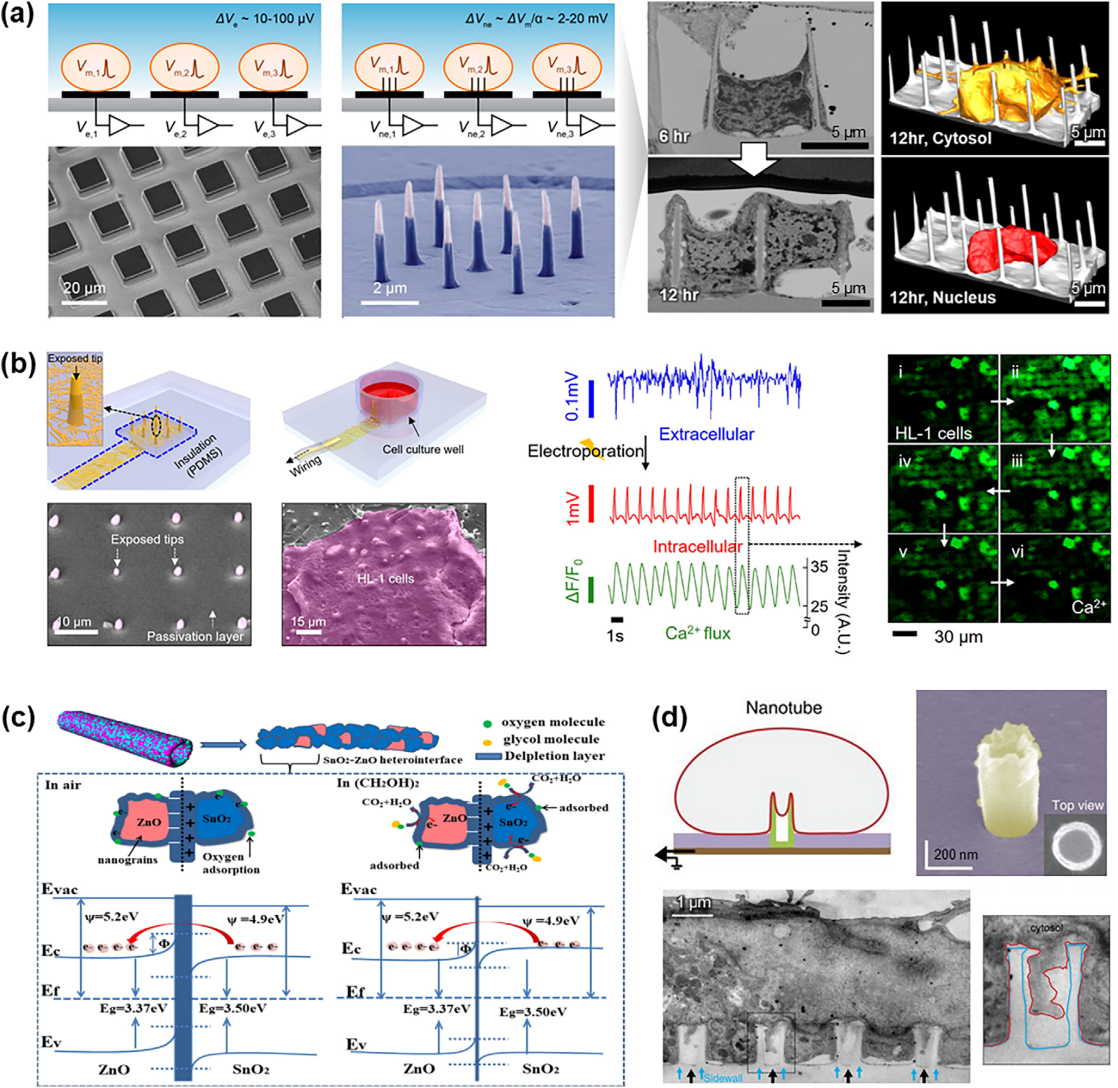
High‐aspect‐ratio nanostructures in biosensing and cell interfacing. a) Planar and nanoneedle‐incorporated electrodes for intracellular measurements, and high‐resolution images demonstrating cellular interfacing capabilities of nanoneedles. Reproduced with permission.^[^ ^11^, ^90^ ^]^ Copyright 2018, American Chemical Society. Copyright 2024, Wiley. b) Vertically ordered silicon nanoneedles on transparent elastomeric substrates, enabling simultaneous intracellular recording and live cell imaging with enhanced electrochemical and optical properties. Reproduced with permission.^[^ ^94^ ^]^ Copyright 2023, American Chemical Society. c) ZnO‐SnO₂ heterostructures enhancing gas sensing and biomolecule interaction through electron transport mechanisms. Reproduced with permission.^[^ ^95a^ ^]^ Copyright 2020, Elsevier. d) Nanotube‐based devices for superior cell‐electrode coupling and long‐term stable intracellular recording. Reproduced with permission.^[^ ^97^ ^]^ Copyright 2014, Springer Nature.

High‐aspect‐ratio nanostructures enhance biosensing sensitivity through increased surface area and quantum effects.^[^ ^88^ ^]^ These structures surpass conventional planar electrodes by offering enhanced analyte accessibility and superior signal transduction efficiency.^[^ ^89^ ^]^ Their unique morphology enables precise biological interfacing for single‐cell analysis, intracellular sensing, and low‐abundance biomarker detection (**Figure** **8**a).^[^ ^11^, ^90^ ^]^

Nanoneedles demonstrate remarkable versatility in biological applications, notably through CRISPR/Cas technology integration for intracellular ATP detection with minimal cell viability impact.^[^ ^91^ ^]^ These structures provide scalable, high‐resolution interfaces for electrophysiological recordings in neurons^[^ ^92^ ^]^ and cardiomyocytes.^[^ ^93^ ^]^ Recent breakthroughs include vertically ordered silicon nanoneedles on transparent elastomeric substrates (Figure 8b),^[^ ^94^ ^]^ combining electrochemical performance with optical transparency and mechanical compliance. However, insertion force variability and long‐term cellular stress remain challenges requiring optimization.

Hollow nanotubes enhance biosensing through improved material transfer and cell‐electrode coupling.^[^ ^95^ ^]^ Heterostructured ZnO‐SnO_2_ composite nanotubes demonstrate exceptional gas sensing capabilities, achieving 5 ppm detection limits for glycol (Figure 8c).^[^ ^95a^ ^]^ The tubular structure also facilitates efficient material transfer,^[^ ^96^ ^]^ enabling accurate intracellular recording. Vertically aligned iridium oxide nanotubes show superior performance in long‐term intracellular recordings with minimal invasiveness (Figure 8d).^[^ ^97^ ^]^ These materials establish robust bioelectronic interfaces with high stability and conductivity, although oxidation susceptibility and long‐term biocompatibility remain challenges for clinical translation. Future developments should focus on enhancing material robustness, as well as optimizing fabrication techniques to facilitate integration into next‐generation biosensing platforms.

### 3.5 Emerging Hybrid Nanomaterials

Hybrid and hierarchical nanostructures integrate diverse nanomaterial features across multiple scales, creating synergistic effects that enhance sensitivity and specificity. The combination of metallic NPs with CNTs exemplifies this approach, improving electron transfer kinetics while maintaining high conductivity.^[^ ^98^ ^]^ This approach particularly benefits electrochemical biosensors,^[^ ^99^ ^]^ achieving highly sensitive and selective biomolecule detection for medical diagnostics and environmental monitoring applications.^[^ ^100^ ^]^

**Figure 9:**
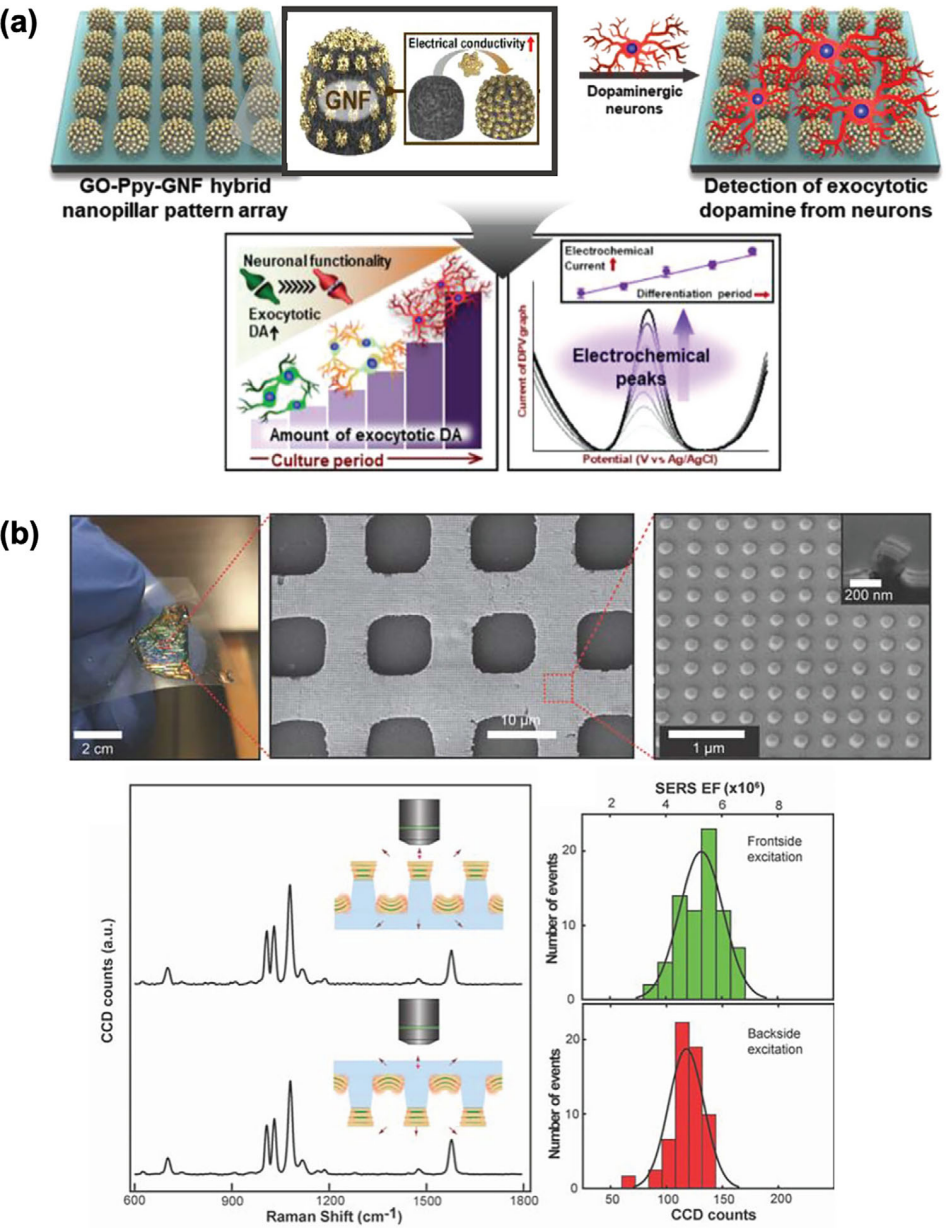
Hybrid nanostructures for biosensing applications. a) Graphene oxide‐incorporated metallic polymer nanopillar array for real‐time electrochemical detection of dopamine exocytosis from SH‐SY5Y cells, assessing dopaminergic neuron functionality. Reproduced with permission.^[^ ^102^ ^]^ Copyright 2023, Wiley. b) Microporous meshes combine polymer meshes with plasmonic nanostructures for enhanced light‐matter interactions, enabling second‐ and third‐harmonic generation and SERS for bacterial biofilm profiling. Reproduced with permission.^[^ ^103^ ^]^ Copyright 2022, Wiley.

Micro‐nano hierarchical structures optimize surface area and cell‐material interfacing.^[^ ^101^ ^]^ Notable examples include graphene oxide‐incorporated metallic polymer nanopillar array for dopamine exocytosis detection from dopaminergic neurons (**Figure** **9**a).^[^ ^102^ ^]^ This platform enables precise assessment of stem‐cell‐derived neuronal functionality, with significant implications for therapeutic applications. Similarly, hierarchical microporous mesh created through micro‐/nanoimprint lithography enhances light‐matter interactions, enabling second‐ and third‐harmonic generation and upconversion photoluminescence for bacterial biofilm profiling (Figure 9b).^[^ ^103^ ^]^

Despite these advances, challenges include complex fabrication processes, limited scalability, and potential material degradation. Future developments must address synthesis optimization, biocompatibility, and long‐term stability for next‐generation biosensing platforms.

## 4 Applying Nanomaterials to the Biosensing Platform

Nanomaterials have transformed biosensing techniques, establishing foundations for innovative and highly sensitive detection methods across diverse biomedical applications. This section reviews primary biosensing techniques utilizing nanomaterials, focusing on their advantages in disease diagnostics, biomarker detection, and personalized medicine.

### 4.1 Electrochemical Biosensors

Electrochemical biosensors demonstrate widespread adoption due to their high sensitivity, rapid response times, and miniaturization potential.^[^ ^104^ ^]^ These sensors excel in complex biological environments, remaining unaffected by light scattering or absorption interference, making them ideal for long‐term monitoring and complex sample analysis. Integrating nanostructures enhances electron transfer kinetics, enabling stronger current responses upon analyte binding.

**Figure 10:**
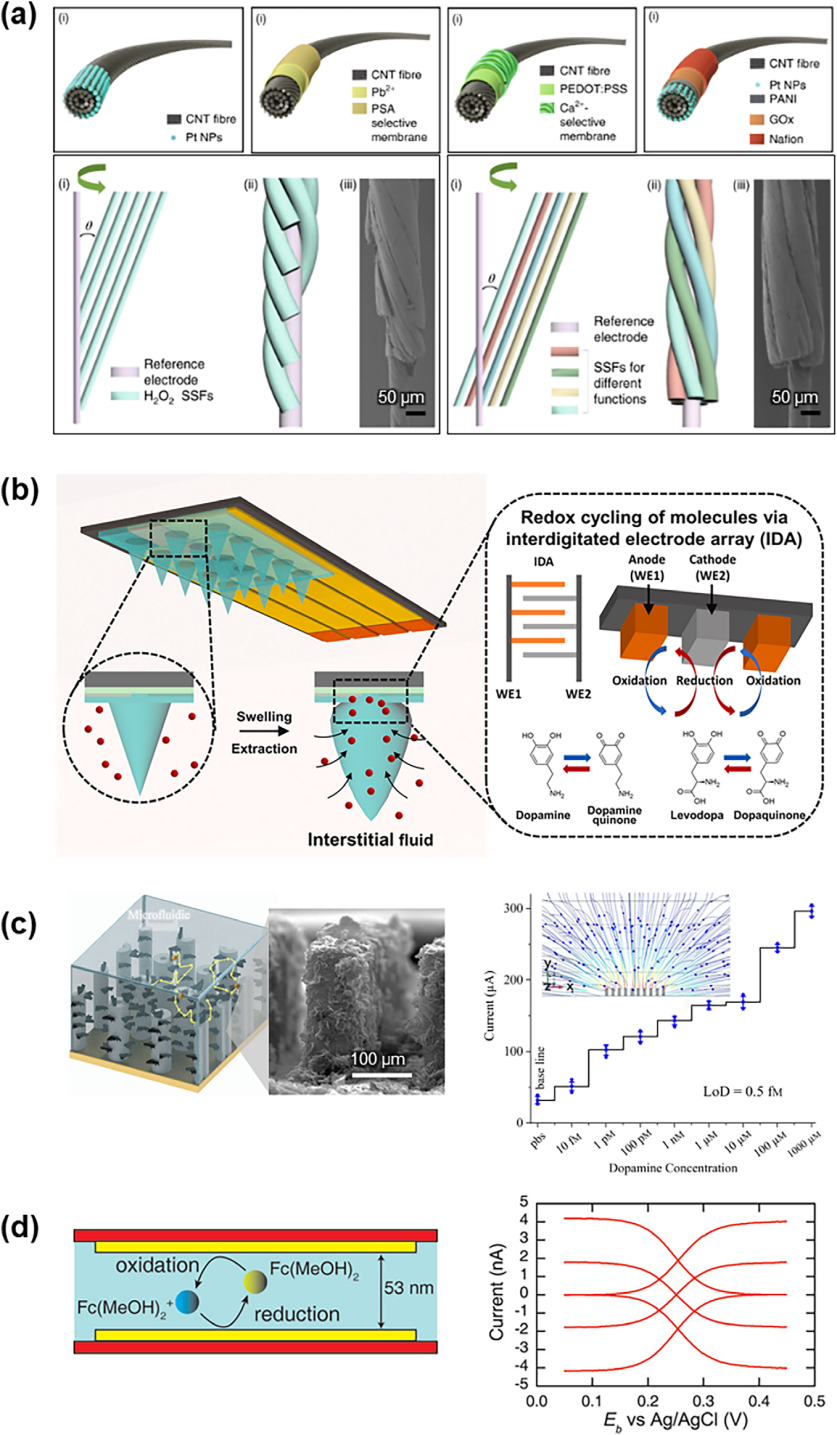
Schematic of advanced nano‐gap sensor devices for in situ biomarker detection. a) Functionalized multi‐walled carbon nanotubes twisted into helical fiber bundles mimic the hierarchical structure of muscle, allowing for real‐time, spatially resolved monitoring of disease biomarkers in vivo. Reproduced with permission.^[^ ^105^ ^]^ Copyright 2020, Springer Nature. b) A hierarchical multi‐length‐scale electrode platform combines graphene‐decorated micropillars for enhanced mass transfer efficiency, achieving attomolar‐level dopamine detection. Reproduced with permission.^[^ ^12a^ ^]^ Copyright 2021, Springer Nature. c) Swellable microneedle‐mounted nanogap sensors detect low concentrations of LDA in interstitial fluid and skin models, enabling non‐invasive real‐time monitoring. Reproduced with permission.^[^ ^106^ ^]^ Copyright 2023, Elsevier. d) Fluidic‐based nanogap sensors with nano‐fluidic channels enhance electrochemical reactions, providing precise, robust in situ analysis for diagnostic applications. Reproduced with permission.^[^ ^108^ ^]^ Copyright 2009, American Chemical Society.

Functionalized multi‐walled CNTs twisted into helical fiber bundles exemplify this advancement, mimicking muscle hierarchical structure for in vivo monitoring of multiple disease biomarkers (**Figure** **10**a).^[^ ^105^ ^]^ These injectable flexible fibers, featuring low bending stiffness and minimal compression stress, enable spatially resolved, real‐time hydrogen peroxide (H₂O₂) detection in tumor‐bearing mice.

Swellable microneedle nanogap sensors address mass transfer limitations (Figure 10b).^[^ ^106^ ^]^ These sensors detect levodopa (LDA), a Parkinson's disease medication, at concentrations of 100 nm in aqueous solutions and 1 µm in skin‐mimicking phantoms and porcine skin, enabling in situ interstitial fluid biomarker detection.

Nanostructured devices with hierarchical designs overcome mass transfer limitations in complex biological fluids. A multi‐length‐scale electrode platform with graphene‐decorated micropillars achieves attomolar‐levels dopamine detection through enhanced mass transfer efficiency (Figure 10c).^[^ ^12a^ ^]^ This demonstrates how hollow and tubular structures efficiently address mass transfer limitations.^[^ ^107^ ^]^

Nano‐fluidic channel integration enables efficient electrochemical reactions within solution‐filled cavity, where nanometer‐scale gaps between parallel electrodes facilitate rapid redox cycling (Figure 10d).^[^ ^108^ ^]^ These fluidic‐based nanogap sensors offer mechanical stability and robustness while enabling precise, real‐time, in situ analysis for advanced diagnostics.

### 4.2 Optical Biosensors

**Figure 11:**
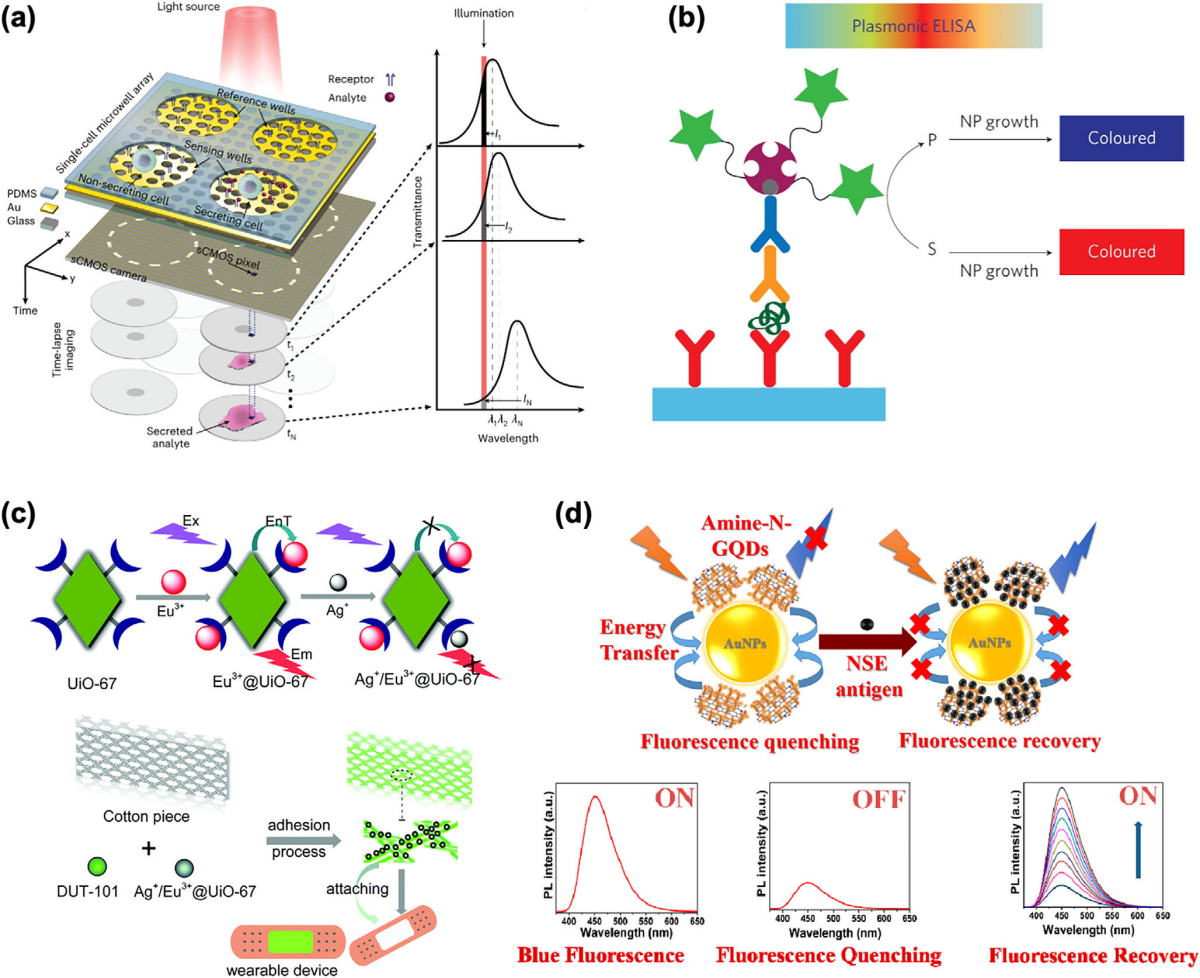
Optical biosensing systems utilizing nanostructures for enhanced sensitivity and specificity. a) A microwell array with nanohole‐patterned gold substrates for real‐time spatiotemporal analysis of single‐cell secretions via SPR detection. Reproduced with permission.^[^ ^67b^ ^]^ Copyright 2023, Springer Nature. b) A colorimetric biosensor combining enzymatic amplification and gold nanoparticle growth to visualize PSA and HIV‐1 p24 at ultra‐low concentrations, suitable for low‐resource settings. Reproduced with permission.^[^ ^15b^ ^]^ Copyright 2012, Springer Nature. c) A wearable sensor utilizing fluorescence detection technology enables real‐time, noninvasive monitoring of chloride (Cl⁻) levels in sweat. Reproduced with permission.^[^ ^113^ ^]^ Copyright 2018, Royal Society of Chemistry. d) A fluorescence turn‐on biosensor using GQDs and AuNPs for neuron‐specific enolase detection, with ultra‐low detection limits for early cancer diagnosis. Reproduced with permission.^[^ ^114b^ ^]^ Copyright 2020, American Chemical Society.

Optical biosensors leverage nanostructures’ unique optical properties for high sensitivity, label‐free biomolecule detection with high spatial resolution.^[^ ^109^ ^]^ SPR‐based sensors enable real‐time monitoring of biomolecular interactions.^[^ ^15^, ^103^, ^110^ ^]^ A microwell array with nanohole‐patterned gold substrates enables spatiotemporal analysis of single‐cell secretions (**Figure** **11**a).^[^ ^67b^ ^]^ Each microwell accommodates individual cells, while receptor‐functionalized nanoholes enhance detection sensitivity for profiling antibody secretion from hybridomas and rare antibody‐secreting human peripheral blood mononuclear cells. This offers valuable insights into protein secretion mechanisms, with applications in immunology and personalized medicine.

For low‐resource settings, enzymatic amplification combined with AuNP growth enables visual detection of prostate‐specific antigen and HIV‐1 capsid antigen p24 at ultra‐low concentrations (1 × 10⁻¹⁸ g mL^−1^) (Figure 11b).^[^ ^15b^ ^]^ Similarly, AuNP‐MOF hybrids serve as SERS detection substrates,^[^ ^111^ ^]^ achieving femtomolar‐level detection of Rhodamine and benzidine through combined plasmonic properties and high adsorption capability. These systems demonstrate high stability and reproducibility for food safety monitoring and clinical diagnostics.

Wearable and portable optical sensors advance real‐time health monitoring.^[^ ^112^ ^]^ A fluorescence‐based device enables noninvasive chloride ion monitoring in sweat (Figure 11c),^[^ ^113^ ^]^ integrating flexible cotton with two lanthanide MOFs (Ag⁺/Eu^3^⁺@UiO‐67 and DUT‐101) as working and reference fluorescence centers.

Nanophotonics^[^ ^114^ ^]^ and microfluidics^[^ ^115^ ^]^ integration enables new capabilities. Biofunctionalized graphene QDs (GQDs) with AuNPs detect neuron‐specific enolase, a small‐cell lung cancer biomarker, via nanosurface energy transfer (Figure 11d).^[^ ^114b^ ^]^ This system achieves an exceptional detection range (0.1 pg mL^−1^ to 1000 ng mL^−1^) with a detection limit of 0.09 pg mL^−1^, highly useful for early cancer diagnostics. A wearable microfluidic nanoplasmonic sensor enables refreshable and portable sweat biomarker detection, incorporating thin plasmonic metasurface with high SERS activity.^[^ ^58^ ^]^

### 4.3 Electrical Biosensors

Electrical biosensors detect analytes through electrical property changes, offering ultra‐fast response and direct electronic system integration. Incorporating nanostructures increases surface area and conductivity, enhancing detection capabilities.

**Figure 12:**
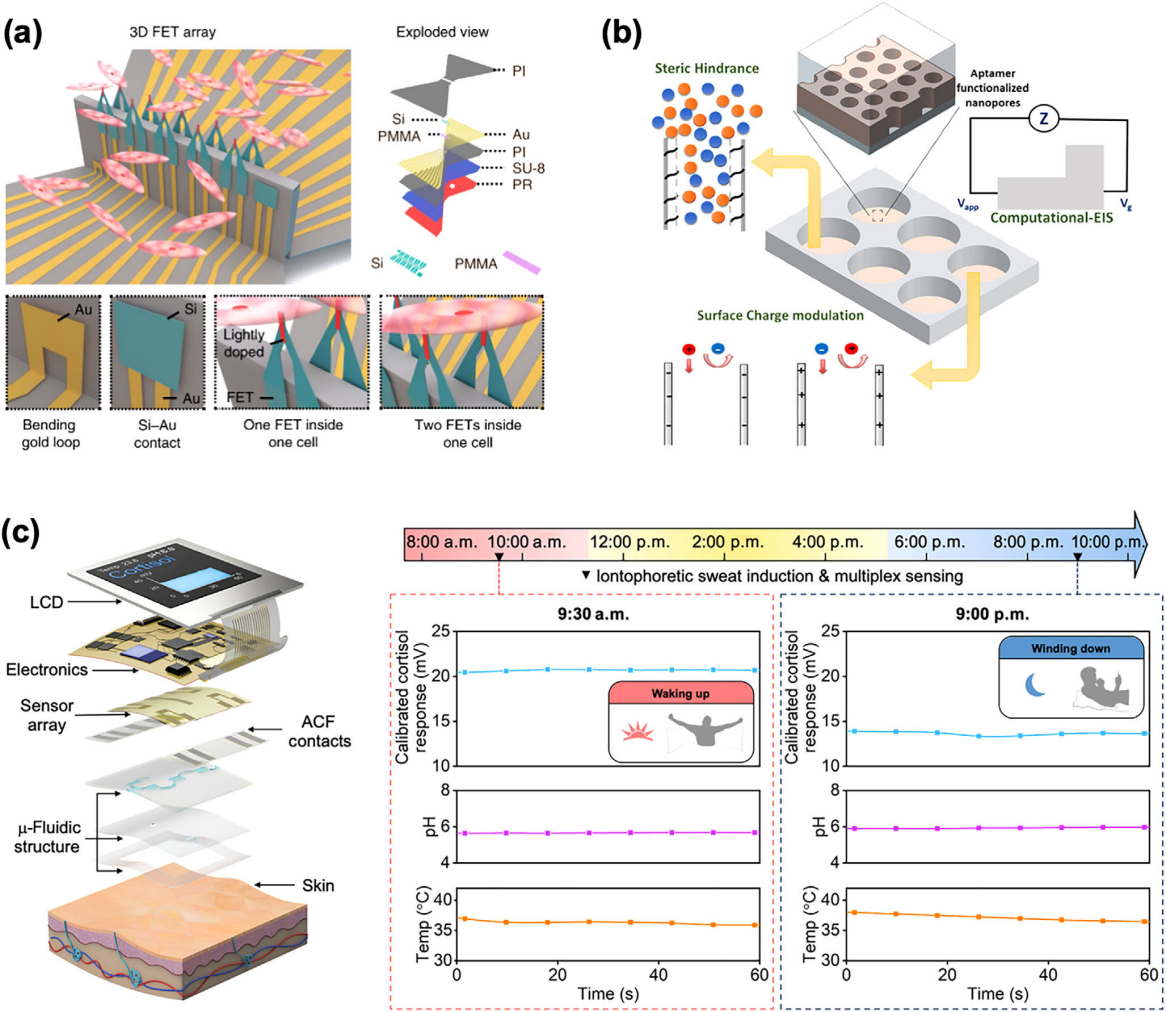
Nanostructure‐enhanced electrical biosensors. a) High‐performance FET array for intracellular signal recording in cardiomyocytes, with high spatial and temporal resolution. Reproduced with permission.^[^ ^116a^ ^]^ Copyright 2022, Springer Nature. b) An impedance biosensor with aptamer‐functionalized nanoporous alumina is used for thrombin detection, achieving high sensitivity. Reproduced with permission.^[^ ^117^ ^]^ Copyright 2022, American Chemical Society. c) Flexible FET biosensor for real‐time cortisol monitoring in sweat, using a cortisol aptamer and In_2_O_3_ FETs. Reproduced with permission.^[^ ^119^ ^]^ Copyright 2022, Science.

FET‐based platforms advance electrophysiology through accurate transmembrane potential recording (**Figure** **12**a).^[^ ^116^ ^]^ 3D FET arrays provide minimally invasive interfaces for intracellular signal conduction velocity measurements in cardiomyocytes with high spatiotemporal resolution. This scalable platform enhances understanding of cellular physiology and pathology through detailed insights into cellular electrical behaviors and interactions.

Nanostructures support charge transfer at electrode interfaces in impedance‐based biosensors for food safety, environmental monitoring, and clinical diagnostics. An aptamer‐functionalized transparent electrode with nanoporous alumina on ITO glass enables label‐free thrombin detection at 10 pm (Figure 12b).^[^ ^117^ ^]^ Combined steric hindrance and surface charge modification enhance sensitivity and signal‐to‐noise ratio, supporting the miniaturization of biosensors.

Electrical biosensors integrate seamlessly with wearable/implantable electronics for real‐time monitoring.^[^ ^118^ ^]^ Nanoscale bio‐receptors modify sensing platforms based on target analyte physicochemical properties. For instance, a FET biosensor array using cortisol‐specific aptamers and nanometer‐thin‐film In₂O₃ FETs enables real‐time stress monitoring (Figure 12c).^[^ ^119^ ^]^ Integrated into smartwatches, this system tracks sweat cortisol levels with high sensitivity for personalized health management.

## 5 Challenges and Future Directions

The integration of nanostructures into biosensing technologies presents several key challenges despite remarkable advancements, particularly for clinical and point‐of‐care applications. Reproducible synthesis of nanomaterials with precise property control remains fundamental. Variations in morphology, size distribution, and surface characteristics significantly impact biosensor performance metrics, including sensitivity, specificity, and reliability. Scalable production of high‐quality nanomaterials while maintaining cost‐effectiveness impedes widespread commercialization.

Beyond materials engineering challenges, biosensor performance in complex biological matrices requires optimization. Signal interference and nonspecific binding in biological fluids compromise sensor sensitivity, particularly for low‐abundance biomarker detection. While aptamer‐based recognition elements have improved specificity,^[^ ^120^ ^]^ distinguishing target signals from background noise remains critical for next‐generation biosensing platforms.

Future developments should focus on multiplexed, miniaturized biosensors for simultaneous multi‐biomarker detection. Integration with digital technologies promises transformed diagnostic capabilities in disease monitoring and personalized medicine.^[^ ^121^ ^]^ Advanced computational methods, including artificial intelligence (AI) and machine learning algorithms, offer promising approaches for real‐time data analysis and adaptive sensor optimization.^[^ ^122^ ^]^ These tools enable sophisticated pattern recognition and improved diagnostic accuracy.

## 6 Conclusion

Integrating nanostructures has transformed biosensing capabilities, enabling unprecedented sensitivity, response speed, and specificity. While nanomaterial science advances continue expanding biomedical applications, successful clinical translation requires addressing key challenges: material reproducibility, biocompatibility optimization, signal processing, and manufacturing scalability.

The convergence of materials engineering, sensor design, and computational analysis advances promise to overcome these barriers, delivering accessible, personalized diagnostic solutions. We hope this review explicitly illuminates both the remarkable progress and persistent challenges, providing foundations for future research that will advance biosensing technology.

## Conflict of Interest

The authors declare no conflict of interest.

## Author Contributions

Y.C. performed conceptualization, data curation, and wrote the original draft. Y.C. performed data curation, wrote the original draft, and visualization. Y.J. performed data curation, wrote, reviewed, and edited the draft, and visualization. H.S. wrote the original draft, wrote, reviewed, and edited the draft, supervision, project administration, and funding acquisition.
